# Perspective and strategy interactively modulate sex differences in a 3D navigation task

**DOI:** 10.1186/s13293-019-0232-z

**Published:** 2019-04-06

**Authors:** TiAnni Harris, Andrea Scheuringer, Belinda Pletzer

**Affiliations:** Department of Psychology and Centre for Cognitive Neuroscience, Paris-Lodron University of Salzburg, Hellbrunnerstraße 34, A-5020 Salzburg, Austria

## Abstract

Sex differences in navigation performance have been attributed to sex differences in information processing during navigation. Perspective refers to the viewpoint of the navigator, with previous work suggesting that men tend to use an allocentric perspective, while women tend to use an egocentric perspective during navigation. Furthermore, different navigation strategies may be used when moving from point A to B, with previous work suggesting that men tend to use a Euclidian strategy, while women tend to use a landmark-based strategy. However, it has not been studied whether perspective and strategy affect sex differences in navigation interactively or independently of each other. The present study aimed to investigate the interactive effects of perspective and strategy on sex differences in a 3D navigation task. In different levels of the task, perspective and strategy were modulated in a 2 × 2 design via different instructions. Potential mediating effects of video gaming experience and sex hormone levels were addressed. We found that men outperformed women in all levels of the navigation task. However, the male advantage was more pronounced using the allocentric perspective compared to the egocentric perspective. When using the allocentric perspective, men showed better performance using a Euclidian strategy while women showed better performance using a landmark-based strategy. The strategy did not modulate performance under the egocentric perspective. Accordingly, sex differences in navigation were interactively modulated by perspective and strategy. These effects were not explained by sex differences in video gaming experience or sex hormone levels.

## Introduction

Virtual navigation tasks have been used to study different forms of navigation [1–3]. Goal-directed navigation, i.e., traveling to a destination, is the most intensely studied form of navigation in humans [4]. According to Spiers and Barry [5], goal-directed navigation depends on three aspects: (i) the properties of the environment itself, (ii) our knowledge about the environment, and (iii) the *navigation strategies* we employ. Virtual navigation tasks have the advantage that they allow us to control the first two aspects, i.e., the properties of the environment and participants’ knowledge of the environment in order to study more explicitly the navigation strategies subjects employ. The term strategy has been used somewhat inconsistently across the navigation literature, and various ways to distinguish different strategies from one another have been utilized [6–8]. The most prominent distinction is probably the distinction between hippocampus-dependent place strategies and caudate-dependent response strategies [8–10]. Note, however, that this distinction has been used in studies on spatial learning, i.e., the strategies are a means to acquire and retrieve information about an environment. Afterward, participants are required to navigate the environment which they got acquainted with. Different terminologies have been used to describe strategies for navigating new environments. These differences vary in linguistic or cartographic information [6, 7]. Nevertheless, there is a consensus on the existence of varying strategies. These strategies differ in how information about the environment is utilized to reach a destination.

Navigating an environment requires two types of spatial information: (i) a sense of direction traveled and (ii) a sense of the distances traveled. Directions can be judged by either an allocentric or an egocentric reference frame [4, 11]. Egocentric navigation judges direction (but also distances) relative to the navigators own position (“left,” “right”), i.e., from the navigators first-person perspective. Allocentric navigation judges direction independent of the navigators’ position, e.g., along cardinal directions (“north,” “south,” etc.). During egocentric navigation, the coordinates of the navigator remain stable, while the coordinates of the target change. During allocentric navigation, on the other hand, the coordinates of the target remain stable, while the coordinates of the navigator change. Distances can be judged metrically or topologically [4]. Metric knowledge involves precise information about the absolute distances (e.g., 35 m) between landmarks in the environment or between the navigator and the target, and is thus often described in Euclidian terms (not to be confused with the Euclidian distance to a target!). Topological knowledge involves less precise information about the relative position between landmarks in the environment or between the navigator and the target. Thus, it is often described in landmark-based terms (e.g., “next to the church”).

These different types of direction (allocentric vs. egocentric) and distance (metric vs. topological/Euclidian vs. landmark-based) information play an important role in the interpretation of sex differences in navigation. While women on average outperform men in verbal and episodic memory tasks, men on average outperform women in visuospatial tasks, like mental rotation, and navigation [12–16]. In fact, navigation is one of the cognitive domains that elicits the most robust sex differences across different cultures [7, 12, 17, 18]. Note that most studies of sex differences in navigation focus on goal-directed navigation in new environments. Men’s precedence in navigation tasks is reflected in fewer navigational errors and faster response times when traveling to a destination. The most robust and consistent sex differences have been found in 3D environments [19–22], while women made fewer errors in 2D navigation tasks [7, 12, 23]. It has thus been claimed that sex differences emerge more consistently in navigation tasks higher in cognitive demands [24, 25]. Furthermore, sex differences in navigation (and other spatial tasks) have been related to training, e.g., higher experience with virtual environments due to video gaming in men [26–28], or hormonal factors, e.g., higher testosterone levels in men ([29–34], but see [21, 35–37]).

Most importantly, sex differences in navigation have been attributed to the use of different navigation strategies by men and women [6, 17, 23, 38, 39]. Various terms have been used to describe these different navigation strategies in men and women (e.g., survey vs. route-based [6], allocentric vs. egocentric [7], Euclidian vs. landmark-based [40]), none corresponding to the terms used for spatial learning (i.e., place vs. response strategies). We here reserve the term “strategy” to distinguish between navigation based on metric (Euclidian) information and landmark-based topological navigation. There is, however, some consistency in the types of spatial information these strategies refer to. During goal-directed navigation, men appear to utilize allocentric and metric information about the environment at a higher rate than women, while women appear to utilize egocentric and topological information at a higher rate than men [6, 7, 41]. This is, for instance, reflected in the way participants remember a map or environment, or the linguistic terms used by participants when describing a path through an environment [17, 23, 38, 42–46]. When participants memorize a map, women recall significantly more landmarks off and on the route, whereas men recall more Euclidian parameters [23, 38, 42, 43]. When participants are asked to describe the path they take, men use more allocentric and Euclidian terms, while women use more egocentric and landmark-based terms [17, 38, 44–46]. Furthermore, men and women differ in their self-reports of navigation strategies. While men report taking shortcuts and focusing on distal landmarks to navigate, women report following well-learned routes and depending on local landmarks [6, 24, 38, 47]. Several studies indicate that women perform better when following egocentric directions, while men perform better when following allocentric directions [6, 7]. Furthermore, multiple studies indicate that women make fewer errors during navigation and show comparable performance to men when more landmark information is available, i.e., in landmark-rich environments [19, 20, 48]. In a 2D matrix navigation task, Saucier et al. [7] phrased directions to a target on the matrix in either allocentric and Euclidian terms or egocentric and landmark-based terms. They observed a male superiority in performance when allocentric and Euclidian terms were used, but a female superiority when egocentric and landmark-based terms were used.

However, so far, no study on sex differences in navigation has studied the two types of information (direction and distance) independently. Rather allocentric directions have been confounded with Euclidian terms to describe distances while egocentric directions have been confounded with landmark-based terms to describe distances [24]. Thus, it remains unclear, whether sex differences in navigation are attributable to the use of different reference frames/perspectives or different strategies to judge distances in the environment. In order to better understand sex differences in navigation strategies, it is thus necessary to dissociate these two types of information. The matrix navigation task developed by Saucier et al. [7] is ideal for that goal.

In a previous study, Scheuringer and Pletzer [40] implemented an adapted version of Saucier’s 2D-version navigation task [21] varying the terms used to describe direction and distance in a 2 × 2 design. However, due to the 2D implementation of the task with north always facing upwards, navigation performance was substantially confounded with mental rotation. Most problematically, participants had to mentally rotate the environment, if egocentric directions were given. Accordingly, Scheuringer and Pletzer [40] observed no female advantage with egocentric directions. In the present study, we employ a 3D adaptation of the Saucier matrix navigation task, but like Scheuringer and Pletzer [40], we use instructions that vary the terms used to describe direction and distance in a 2 × 2 design.

We hypothesize that sex differences in navigation performance depend on the phrasing of directions, in such a way that:

1. Sex differences favoring males will be larger with allocentric directions compared to egocentric directions since allocentric directions force participants to obtain an allocentric perspective, while egocentric directions force participants to obtain an egocentric perspective.
2. Sex differences favoring males will be larger when distances are described in Euclidian terms compared to landmark-based terms since Euclidian terms force participants to use a more metric representation of the environment (Euclidian strategy), while landmark-based terms allow participants to use a topological representation (landmark-based strategy).

Accordingly, we expect the largest sex differences in the condition combining allocentric directions with Euclidian terms (interactive effect). Since previous studies have attributed sex differences in navigation to training or hormonal factors [35–38], we additionally address potential mediating effects of video gaming experience and sex hormone levels.

## Method

### Participants

Eighty-four healthy participants (42 men; 42 women), aged 18–36 years, were recruited for this study. Exclusion criteria were physical, endocrine, and mental illness; hormonal contraception or medication; and left-handedness. A regular menstrual cycle [49] was required for female participants, ranging from 21 to 35 days. Previous studies demonstrate that navigation strategies also vary along the menstrual cycle with increased use of landmark information during the high-hormone luteal cycle phase [40, 50]. Accordingly, in the present study, all women were tested during their mid-luteal cycle phase, which was determined as follows: Based on participants’ self-reports about the onset of last period and cycle duration, ovulation was calculated 14 days before the assumed onset of their next period. The testing session was scheduled 3–10 days after ovulation, i.e., 10–3 days before the expected onset of the next menses. Three women were excluded, because of extremely low progesterone values (< 48 pg/ml, see below), suggesting a mismatch between the calculated cycle phase and hormone values. Furthermore, one man was excluded due to estradiol values exceeding the group mean by more than three standard deviations (SD). This resulted in a final sample of 80 participants, 41 men (mean age 24.29, SD = 2.40, range 20–29 years) and 39 women (mean age 23.17, SD = 4.97, range 18–36 years) with an average cycle duration of 29.19 days (SD = 2.79 days), who were on average tested on day 22.23 (SD = 3.53) of their menstrual cycle. Age did not differ significantly between men and women (*t* = 1.30, *p* = .197), neither did education (*t* = − 1.22 *p* = .226) nor IQ (*t* = .890, *p* = .376; men: mean IQ 108.36, SD = 9.18; women: mean IQ 105.10, SD = 21.70). All participants had a minimum of 8 years of secondary education and had passed the general qualification for university entrance.

### Ethics statement

All participants gave their informed written consent to participate in the study. The methods used conformed to the Code of Ethics of the World Medical Association (Declaration of Helsinki).

The institutional guidelines of the University of Salzburg (Statutes of the University of Salzburg^1^) state in §163 (1) that it is necessary to seek ethical approval for research on human subjects if the physical or psychological integrity is affected, the right for privacy or other important rights or interests of the subjects or their dependents are confounded. Paragraph §163 (2) states that it is the decision and responsibility of the PI to decide whether (1) applies to a study or not. Therefore, no ethical approval for this study was sought out. Non-invasive methods were used on healthy adult volunteers, who willingly gave their informed consent to participate. Accordingly, (1) did not apply. Data were processed in anonymized/de-identified form. Participants were assigned a subject ID (v001, v002, etc.), when physically present at the lab, which was used throughout the study.

### Navigation task

**Fig. 1:**
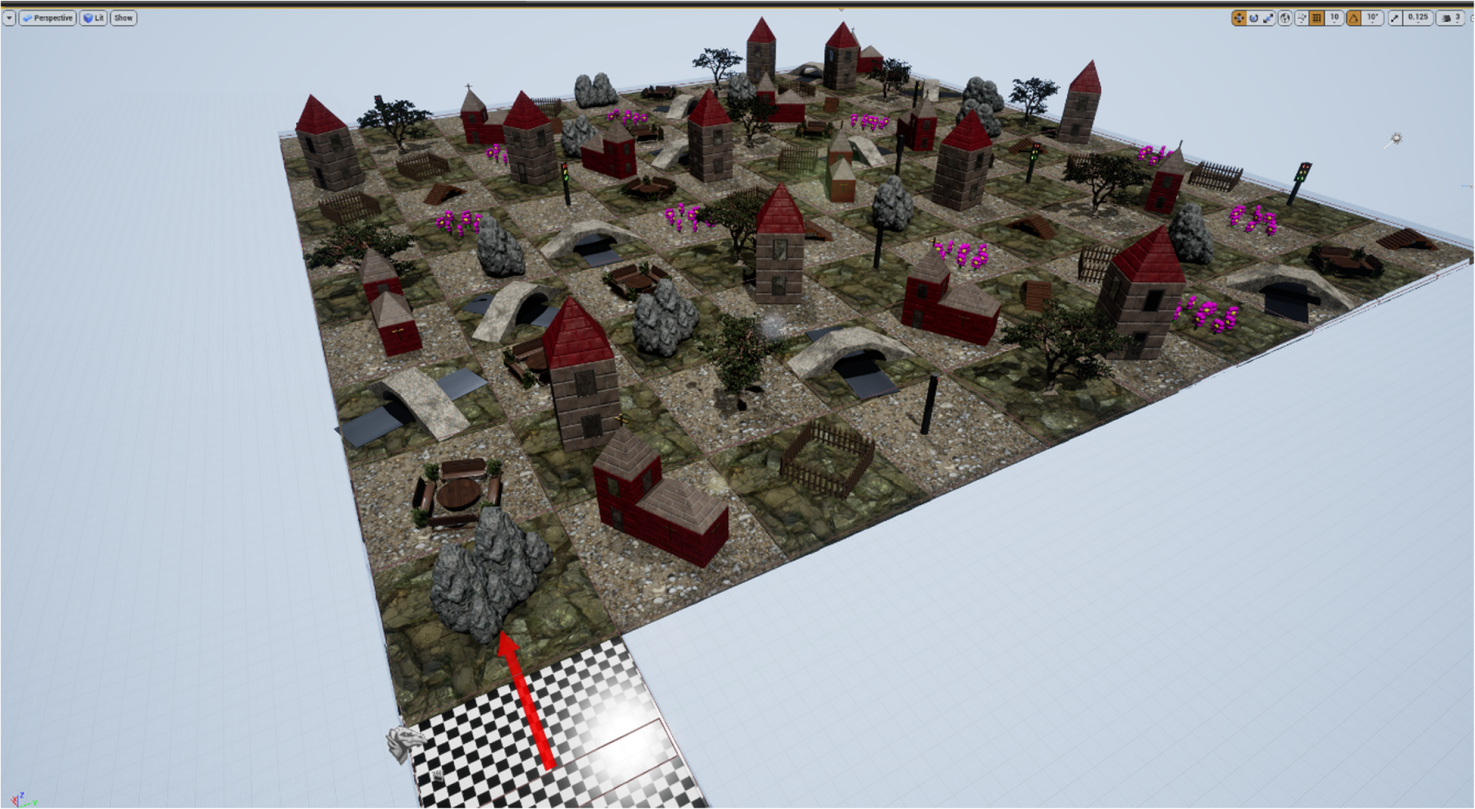
Layout of an exemplary level of the navigation task. Start field with an arrow indicating the cardinal direction (bottom left corner). 10 × 10 matrix with each item appearing once in each row and column

**Fig. 2:**
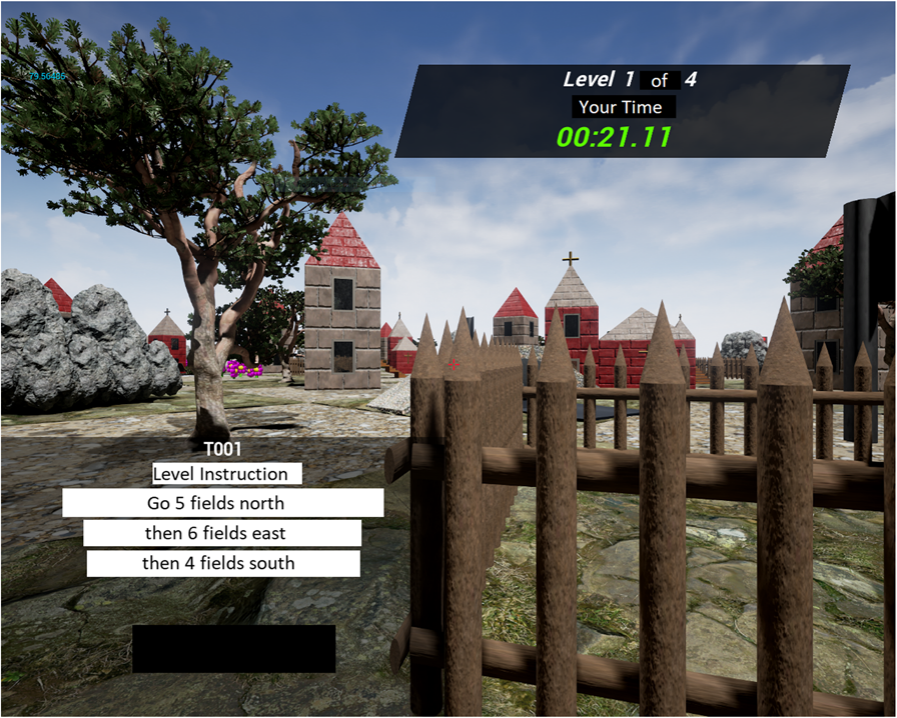
Example of directions for one level of the navigation task. The instructions were presented in the bottom left corner; a participant is navigating through the virtual world. Examples of items placed in the world are visible such as fence, tree, house, church, boulders, and flowers

For this study, 20 task environments of a 3D navigation task were created using Unreal Engine 4 Version 8.1, building on a 2D version employed by Scheuringer and Pletzer [40], which was adapted from Saucier et al. [7]. Each task environment was a checkerboard consisting of 100 squares (10 × 10) plus 1 starting square positioned on the outside (Fig. 1). One of the ten real-life landmarks (tree, bridge, stairs, house, church, bench, boulder, street light, fence, flowers) was placed in each square. The order of landmarks was different for each task environment. However, in each task environment, each item only appeared once in each row and column, meaning each item could be found ten times in each task environment. That way, rows and columns share similarities with streets which participants may encounter in real life with the exception that each landmark occurs only once in each row/column. This restriction is necessary to not confound Euclidian and landmark-based distance information (see below). Accordingly, each task environment was new to the participants, similar to a new city or a new part of town. In each level of the navigation task, participants had to navigate one of these new environments. Each level started with a countdown (“three, two, one, go”), while participants were positioned on the starting square facing the new environment. After the countdown, participants used the arrow keys to navigate the environment. Their task was to reach a target location following a path, which was indicated by three lines of instructions (Fig. 2). Four conditions were characterized by different phrasings of the instructions, which modulated the terms to describe direction (here: perspective) and the terms to describe distances (here: strategy) in a 2 × 2 design: *allocentric Euclidian* (“go east for 4 blocks”), *allocentric landmark-based* (“go east until you reach the tree”), *egocentric Euclidian* (“turn right and go for 4 blocks”), and *egocentric landmark-based* (“turn right and go until you reach the tree”). There were four training levels, i.e., one for each condition. Table 1 lists example directions for each condition. After the training, participants completed 16 task levels, i.e., 4 task levels per condition. The order of conditions was pseudo-randomized throughout the task. At the beginning of each level, i.e., after the countdown, participants were informed which cardinal directions they were currently facing. Starting directions were counterbalanced across conditions, i.e., among the four items per condition, one started facing north, one facing south, one facing east, and one facing west. Accordingly, participants started four times in each cardinal direction. Each path encompassed 15 squares and 2 turns. Without moving backward, there were four possible patterns of movement in the environment: two left turns or two right turns resulted in a U-shaped path, and a left turn followed by a right turn or a right turn followed by a left turn resulted in a Z-shaped path. The pattern of movement, as well as the number of squares before the first and second turn, was counterbalanced across conditions to ensure that participants could not predict the path based on experience from previous levels. Participants only advanced to the next level when the target location was reached and the correct location was confirmed by pressing the space bar. When the final destination of each level was successfully reached, and the space bar was pressed, participants had to identify north. A screen with four arrows pointing toward each edge of the screen appeared. By pressing the arrow keys on their keyboard, participants could indicate where they assumed north was. There were no maximum time restrictions for the navigation nor the north question.

**Table 1:** Training level directions

	Perspective	
	Allocentric	Egocentric
Strategy		
Euclidian	Go east for 4 blocks	Go straight ahead for 4 blocks
	Then go north for 5 blocks	Then turn left and go 5 blocks
	Then go east for 6 blocks	Then turn right and go 6 blocks
Landmark	Go east until you reach the flower	Go straight ahead until you reach the flower
	Then go north until you reach the bridge	Then turn left until you reach the bridge
	Then go east until you reach the fence	Then turn right until you reach the fence

The measures of performance *navigation time* in seconds (navigation time—NT) was recorded by timing the participant from the moment the “go” appeared up to the moment the space bar was activated at the correct location. *Accuracy* (was the correct arrow pressed which represents north? Yes or no was measured) and *reaction time* in seconds, from the moment the identifying north screen appeared until an arrow key was pressed, were also recorded.

In order to correctly identify north after reaching the target location, it was required to consider all turns made since starting. It was assumed that navigation time reflected navigation abilities, while performance in identifying north reflected mental rotation abilities when using different perspectives and strategies.

### Procedure

Participants completed the 3D navigation task as part of a larger study. Participants were asked not to eat, drink, or smoke for 30 min before arriving at the laboratory. When participants arrived at the computer lab of the University of Salzburg, they were asked to rinse out their mouths and fill out the informed consent form and a screening questionnaire pertaining to the exclusion criteria and habits (sleeping, eating, smoking, etc.). Afterward, i.e., approximately 15 min after arriving at the lab, they gave the first of three saliva samples and completed two computerized tasks addressing attention and numerical processing, respectively. These two tasks, which took approximately 30 min, were always completed in the same order and will be described elsewhere. Following this, the second saliva sample was taken for sex hormone analysis. Then, the 3D navigation task was completed by each participant, followed by the last saliva sample. The duration of the navigation task varied across participants depending on their performance but took approximately 30 min. In the end, participants indicated their computer gaming experience on a scale of 1–9. They were specifically instructed to indicate how much experience they had with ego-shooter video games, 1 meaning no experience at all and 9 being professional gamers. Furthermore, participants completed the ten-item APM screening (advanced progressive matrices [51]) as implemented in the Vienna Test System [52] as a coarse measure of general intelligence to ensure that there were no substantial differences in IQ between men and women and that all participants had sufficient cognitive abilities to recognize, process, and handle new patterns as presented during the navigation task. Participants were debriefed and received either course credit or 10 € for participating.

### Hormone analyses

Each participant gave three saliva samples (compare the “Procedure” section). Saliva samples were stored at − 20 °C and centrifuged twice at 3000 rpm for 15 and 10 min, respectively, prior to hormone assessment. The three samples of each participant were pooled before hormone analysis in order to assess average hormone concentrations over the whole session, thereby controlling for fluctuations related to salivary production and ensuring the reliability of hormone assessment. Testosterone, 17β-estradiol, and progesterone were assessed from the pooled sample of each participant using salivary ELISA kits by DeMediTec.

Participants with hormone values deviating by more than three standard deviations from the group mean were excluded. In addition, we expected progesterone values for women to fall within a normal range for the luteal cycle phase. As recommended by the DeMediTec kit instructions, we established this range for our laboratory based on an unrelated sample of 60 women tested with the DeMediTec salivary progesterone ELISA kit in three menstrual cycle phases (compare Pletzer et al., accepted). In all women, progesterone levels were higher during the luteal phase compared to the other cycle phases. While there was substantial variation in the luteal phase values, none of the women displayed a luteal progesterone value below 48 pg/ml. Taking into account the assay sensitivity of 5 pg/ml, a progesterone cutoff of 43 pg/ml was established for inclusion in a luteal phase sample.

### Statistical analyses

Navigation time (NT) and accuracy, as well as reaction time when identifying north, were analyzed in the context of linear mixed effects models (lmes) using the *lmer* function of *lme4* package (Version 1.1-18-1) [53] of statistics software RStudio 1.1.456 In all models, the participant number was modeled as a random factor. Furthermore, backward elimination of non-significant interactions was applied to all models using the *step* function of the *lmerTest* package (Version 3.0-1). The following models were evaluated:

In a first step, we addressed sex differences in the dependent variables and their modulation by perspective and strategy by introducing the interactive effect of sex*perspective*strategy as a fixed effect in the model. To control for training effects, a level was additionally entered as a covariate in the model (e.g., NT ~ 1|PNr + level + sex*perspective*strategy). If significant sex differences were found, potential mediating effects of ego-shooter gaming experience were addressed in a second step using three models. First, it was established whether sex affected ego-shooter experience (ego-shooter ~ sex). Second, it was established whether ego-shooter experience affected navigation time (NT ~ 1|PNr + ego-shooter*perspective*strategy). Third, both sex and ego-shooter experience were entered in the model (NT ~ 1|PNr + sex*ego-shooter*perspective*strategy). In the third step, the same procedure was used to assess potential mediating effects of sex hormone levels.

In all models, both, the dependent and continuous independent variables were *z*-standardized using the *scale* function. Therefore, the coefficients *b* of fixed effects in the models represent a standardized effect size based on standard deviations, similar to Cohen’s *d*.

### Data availability

The datasets generated and/or analyzed during the current study are available from the corresponding author upon reasonable request.

## Results

### Sex differences in navigation time and their modulation by perspective and strategy

To evaluate sex differences in navigation performance and whether they were modulated by *perspective* and *strategy*, we applied a lme on the dependent variable navigation time modulating participant number as a random factor and level, as well as the interactive effects of sex*perspective*strategy as fixed effects (NT ~ 1|PNr + level + sex*perspective*strategy).

Level had a significant negative effect on navigation time (*b* = − 0.10, SE_b_ = 0.03, *t*_(1216)_ = − 3.88, *p* < .001). Participants completed the levels faster the more levels they had completed.

**Fig. 3:**
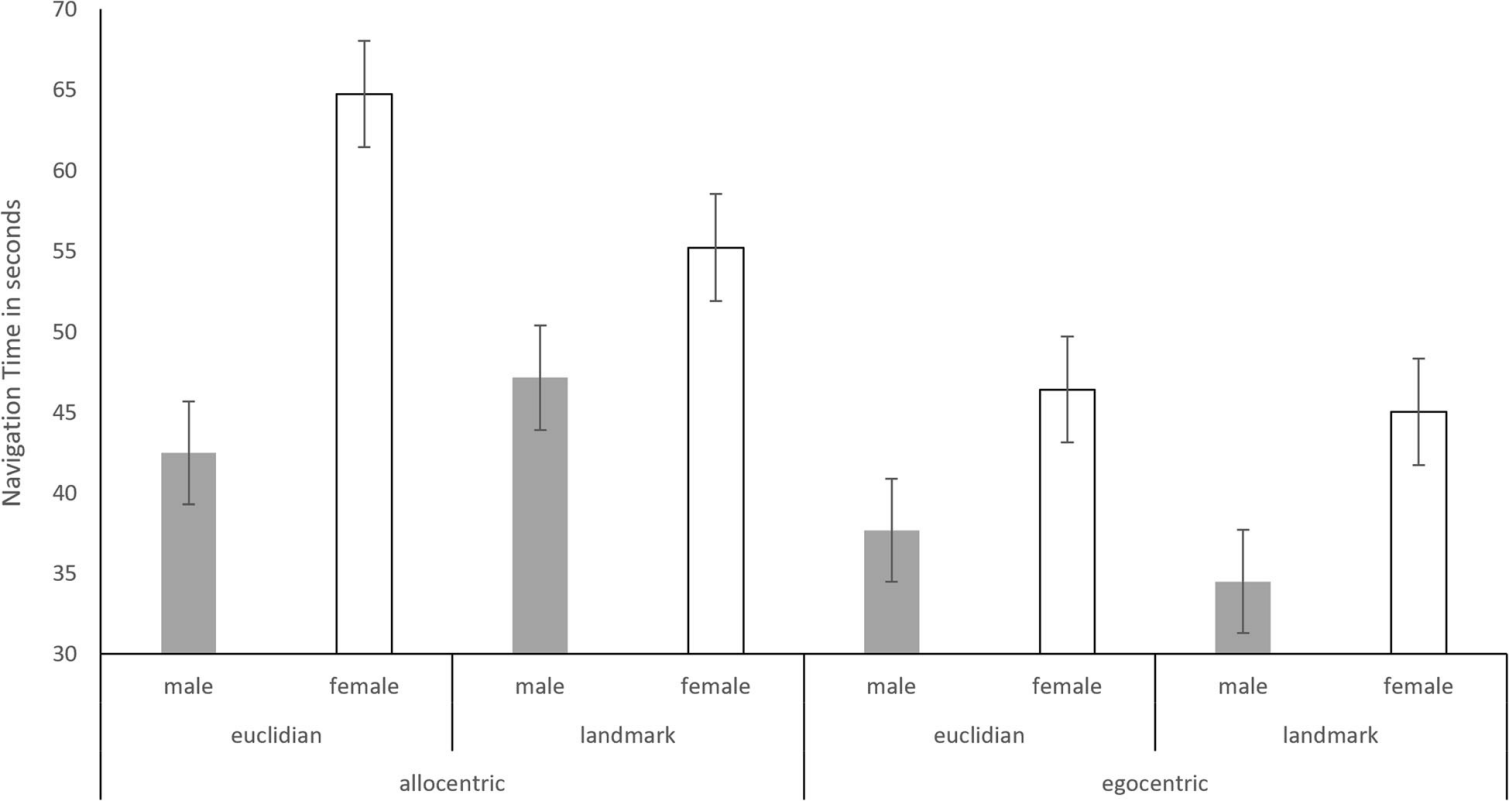
Navigation time for all groups and conditions. A threefold interaction between sex, perspective, and strategy was observed. Men reach the target location faster than women in all conditions. Both men and women reach the target location faster with egocentric directions compared to allocentric directions. The effect of perspective is larger in women compared to men. No strategy differences in performance were observed for the egocentric perspective. For the allocentric perspective, men reached the target location faster with the Euclidian strategy, while women reached the target location faster with the landmark-based strategy

The main effects of sex were significant (*b* = 0.60, SE_b_ = 0.12, *t*_(80)_ = 4.86, *p* < .001). Men showed a faster navigation time (40.46 s) compared to women (52.82 s). The main effects of perspective (*b* = − 0.13, SE_b_ = 0.10, *t*_(1216)_ = − 1.29, *p* = .196) and strategy (*b* = 0.13, SE_b_ = 0.10, *t*_(1216)_ = 1.26, *p* = .205) as well as the perspective*strategy interaction (*b* = − 0.21, SE_b_ = 0.14, *t*_(1216)_ = − 1.49, *p* = .136) did not reach significance. Sex showed significant interactions with both perspective (*b* = − 0.37, SE_b_ = 0.14, *t*_(1216)_ = − 2.56, *p* = .011) and strategy (*b* = − 0.38, SE_b_ = 0.14, *t*_(1216)_ = − 2.69, *p* = .007). Furthermore, there was a significant three-way interaction of sex*perspective*strategy (*b* = 0.43, SE_b_ = 0.20, *t*_(1216)_ = 2.14, *p* = .033) (Fig. 3).

In order to resolve these interactions, separate models were run for men and women (NT ~ 1| PNr + perspective*strategy), for the allocentric and egocentric perspective (NT ~ 1| PNr + sex*strategy), and for the Euclidian compared to the landmark-based strategy (NT ~ 1|PNr + sex*perspective). For both men and women, a significant main effect of perspective was observed (men: *b* = − 0.25, SE_b_ = 0.10, *t*_(627)_ = − 2.47, *p* = 0.014; women: *b* = − 0.48, SE_b_ = 0.10, *t*_(590)_ = − 4.64, *p* < 0.001), indicating faster navigation with egocentric compared to allocentric directions. The effect size was much larger in women. The main effect of strategy was only significant in women (*b* = − 0.22, SE_b_ = 0.10, *t*_(590)_ = − 2.08, *p* = .038), indicating faster navigation with a landmark-based strategy compared to the Euclidian strategy. In men, the main effect of strategy did not reach significance, but the effect size indicates faster navigation time with the Euclidian compared to the landmark-based strategy (*b* = 0.17, SE_b_ = 0.10, *t*_(627)_ = 1.65, *p* = 0.100). The perspective*strategy interaction did not reach significance in either men or women (all |*b*| < 0.25, all SE_b_ < 0.14, all *t*_(627)_ > 1.74, all *p* < 0.082).

For both perspectives, the main effect of sex was significant (allo: *b* = 0.60, SE_b_ = 0.16, *t*_(80)_ = 3.71, *p* < 0.001; ego: *b* = 0.23, SE_b_ = 0.08, *t*_(80)_ = 2.94, *p* = 0.004), indicating faster navigation time for men compared to women. The effect size of sex differences was larger for allocentric compared to the egocentric directions. The main effect of strategy was non-significant (both |*b*| < 0.10, both SE_b_ > 0.09, both |*t*| < 1.01, both *p* > 0.31). However, we observed a significant interaction of sex*strategy with allocentric directions (*b* = − 0.38, SE_b_ = 0.19, *t*_(570)_ = − 2.06, *p* = .040), which was absent with the egocentric directions (*b* = 0.05, SE_b_ = 0.07, *t*_(567)_ = 0.72, *p* = .470). With allocentric directions, women showed significantly faster navigation times for the landmark compared to the Euclidian strategy, while men showed faster navigation times for the Euclidian compared to the landmark strategy.

For both strategies, navigation time was faster for men compared to women (eucl: *b* = 0.60, SE_b_ = 0.13, *t*_(80)_ = 4.55, *p* < 0.001; landm: *b* = 0.22, SE_b_ = 0.11, *t*_(80)_ = 1.96, *p* = 0.053). The effect size of sex differences was larger for the Euclidian compared to landmark strategy. Navigation time was faster for egocentric compared to allocentric perspective in both strategies (both |*b*| > 0.20, both SE_b_ > 0.08, both |*t*| > 1.78, both *p* < 0.07). However, we observed a significant interaction of sex*perspective in the Euclidian strategy (*b* = − 0.36, SE_b_ = 0.16, *t*_(568)_ = − 2.29, *p* = .02), which was absent in the landmark strategy (*b* = 0.06, SE_b_ = 0.13, *t*_(569)_ = 0.49, *p* = .617). Sex differences were larger for allocentric compared to egocentric directions only with the Euclidian strategy.

### The mediating role of ego-shooter experience on sex differences in navigation time

In order to evaluate whether the sex influence on navigation performance was mediated by *ego-shooter experience*, the following steps were performed.

In the first step, we evaluated sex differences in ego-shooter experience. Sex had a significant effect on ego-shooter experience (*b* = − 1.17, SE_b_ = 0.18, *t*_(79)_ = − 6.40, *p* < .001), indicating higher ego-shooter experience in men compared to women.

In the second step, we evaluated the impact of ego-shooter experience on navigation performance. To that end, we applied a lme on the dependent variable navigation time modulating participant number as a random factor and level, as well as the interactive effects of egoshooter*perspective*strategy as fixed effects (NT ~ 1|PNr + level + egoshooter*perspective*strategy).

There were significant main effects of ego-shooter experience (*b* = − 0.22, SE_b_ = 0.05, *t*_(79)_ = − 4.40, *p* < .001) and perspective (*b* = − 0.31, SE_b_ = 0.05, *t*_(1205)_ = − 6.04, *p* < .001). The main effect of strategy did not reach significance and showed no significant interaction with perspective or ego-shooter experience. It was thus removed from the model. There was, however, a significant interaction between ego-shooter experience and perspective (*b* = 0.11, SE_b_ = 0.05, *t*_(1205)_ = 2.07, *p* = .038), indicating a smaller impact of the perspective effect in participants with larger ego-shooter experience.

In the third step, we evaluated, whether ego-shooter experience could explain the impact of sex on navigation performance, by entering both variables as fixed effects into a linear mixed model (NT ~ 1|PNr + level + sex*egoshooter*perspective*strategy).

In this model, the main effect of ego-shooter experience did not reach significance and did not interact with any other factor. It was thus removed from the model, resulting in a final model as described in the “Sex differences in navigation time and their modulation by perspective and strategy” section. Thus, rather than the sex difference in navigation performance being mediated by ego-shooter experience, the effect of ego-shooter experience on navigation performance is mediated by sex.

### The mediating role of sex hormones on sex differences in navigation time

To evaluate whether sex influences on navigation performance were mediated via sex hormone levels, the following analyses were performed.

In the first step, we confirmed that men and women differed in their sex hormone levels. Men had significantly higher *testosterone* levels compared to women (*b* = − 1.19, SE_b_ = 0.18, *t*_(76)_ = − 6.42, *p* < .001), whereas women had significantly higher *progesterone* levels (*b* = 1.04, SE_b_ = 0.20, *t*_(75)_ = 5.26, *p* < .001) and, by trend, higher *estradiol* levels (*b* = 0.44, SE_b_ = 0.23, *t*_(75)_ = 1.95, *p* = .055) compared to men.

In the second step, we evaluated the impact of sex hormones on navigation performance. To that end, we applied lmes on the dependent variable navigation time modulating participant number as a random factor and level as well as the interactive effects of hormone*perspective*strategy as fixed effects (NT ~ 1|PNr + level + hormone*perspective*strategy). Testosterone and progesterone were significantly related to navigation time (testosterone: *b* = − 0.10, SE_b_ = 0.05, *t*_(76)_ = − 2.17, *p* = 0.034; progesterone: *b* = 0.12, SE_b_ = 0.05, *t*_(75)_ = 2.54, *p* = 0.013), but did not interact with perspective and strategy. Time to reach the target was lower in participants with higher testosterone and lower progesterone levels. Estradiol was not related to navigation time and did not interact with perspective and strategy; thus it was removed from the model.

In the third step, we evaluated whether sex hormone levels could explain the impact of sex on navigation performance by entering both variables as fixed effects into a linear mixed model (NT ~ 1|PNr + level + sex*hormone*perspective*strategy). In this model, none of the hormones showed a significant main effect and none of them interacted with any other factor. They were thus removed from the model. Thus, rather than the sex difference in navigation performance being mediated by sex hormone levels, the effects of sex hormone levels on navigation performance are mediated by sex.

### Identifying north: accuracy and reaction time

In order to evaluate sex differences in the speed and accuracy of identifying north and whether it was modulated by perspective and strategy, we applied lmes on the dependent variables *accuracy* and *reaction time* modulating participant number as a random factor and the interactive effects of sex*perspective*strategy as fixed effects (north ~ 1|PNr + level + sex*perspective*strategy).

In both models, the main effect of sex was non-significant and did not interact with perspective or strategy. It was thus removed from the models. Level had a significant effect on both accuracy (*b* = 0.10, SE_b_ = 0.03, *t*_(1221)_ = 3.28, *p* = .001) and reaction time (*b* = − 0.27, SE_b_ = 0.03, *t*_(1220)_ = − 8.76, *p* < .001). Participants rated north more accurately and faster the more levels they had completed.

**Fig. 4:**
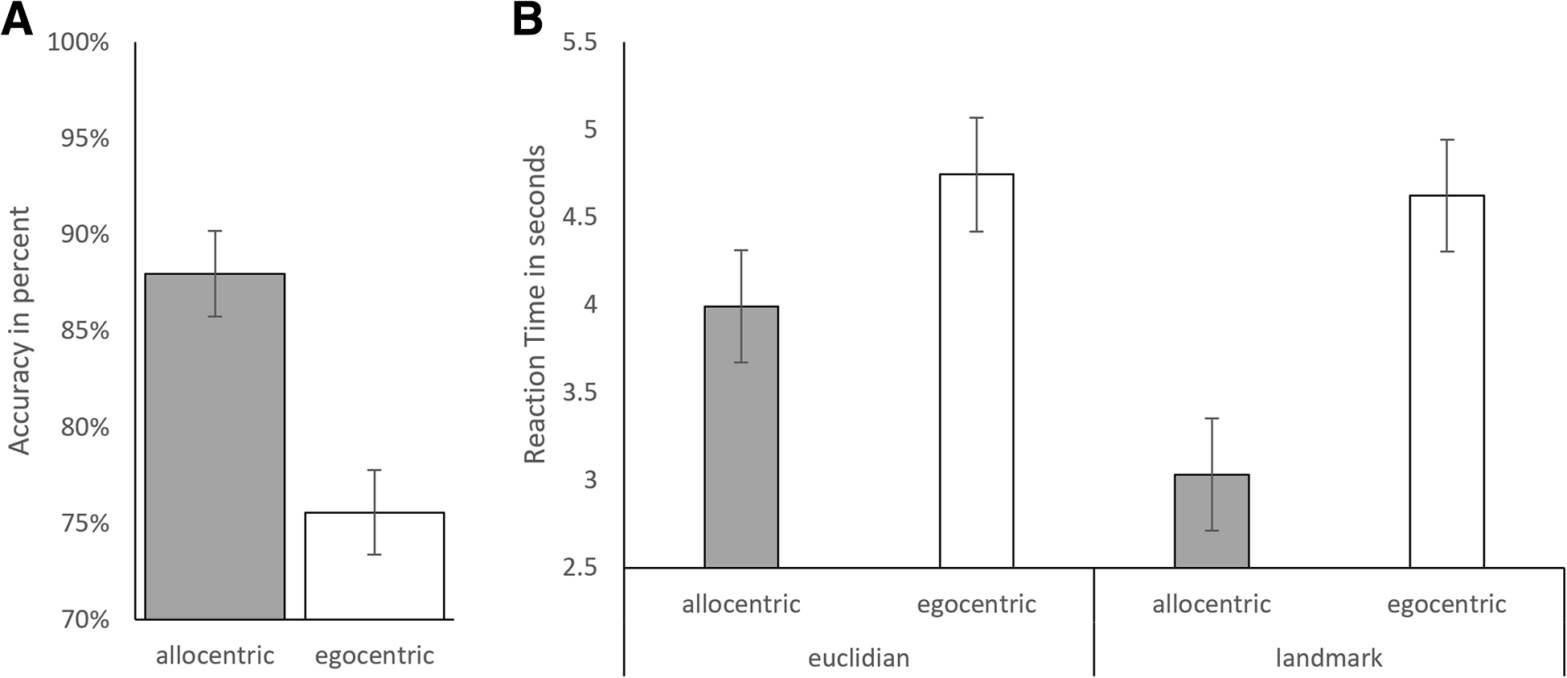
Accuracy and reaction time in identifying north. **a** *Accuracy*: Accuracy in identifying north was significantly higher for the allocentric perspective compared to the egocentric perspective. No effect of strategy was observed. **b** *Reaction time:* The landmark strategy resulted in faster identifications for the allocentric perspective, whereas the Euclidian strategy resulted in faster identifications for the egocentrical perspective. Men and women did not differ in accuracy and reaction time when identifying north

In the analysis of accuracy, the main effect of perspective (both *b* = − 0.32, SE_b_ = 0.05, *t*_(1221)_ = − 6.41, *p* < .001) was significant, indicating a higher accuracy with the allocentric (89%) compared to the egocentric perspective (76%). The main effects of sex and strategy, as well as all interactions, did not reach significance and were thus removed from the model (Fig. 4a).

In the analysis of reaction time, the main effect of perspective (*b* = 0.25, SE_b_ = 0.05, *t*_(1220)_ = 5.18, *p* < 0.001) and strategy were significant (*b* = − 0.12, SE_b_ = 0.05, *t*_(1220)_ = − 2.41, *p* = 0.016). The interaction between strategy and perspective was non-significant and thus removed from the model. These results indicate that north was identified faster with allocentric compared to egocentric directions and with the landmark compared to the Euclidian strategy (Fig. 4b).

Since no sex differences were observed in the ability to correctly identify north, modulating roles of ego-shooter experience and sex hormones were not addressed further.

## Discussion

The present study aimed to investigate whether sex differences are modulated by strategy and perspective during a 3D navigation task. Since the allocentric perspective and Euclidian strategy both incorporate a more global view on navigation and egocentrical perspective and landmark-based strategy a more localized view on navigation, the following hypotheses were established:

1. Sex differences favoring males will be larger with allocentric directions compared to egocentric directions since allocentric directions force participants to obtain an allocentric perspective while egocentric directions force participants to obtain an egocentric perspective.
2. Sex differences favoring males will be larger when distances are described in Euclidian terms compared to landmark-based terms since Euclidian terms force participants to use a more metric representation of the environment (Euclidian strategy), while landmark-based terms allow participants to use a topological representation (landmark-based strategy).

Interactions between perspective and strategy were explored. Furthermore, we address the potential mediating effects of video gaming experience and sex hormone levels.

We observed that men find the target location significantly faster across all strategies and perspectives than women. This is in line with multiple previous findings, demonstrating a male superiority in spatial navigation [19–22]. It is, however, in contrast with previous findings, suggesting that a landmark-based strategy or egocentric perspective can reduce or reverse the sex difference in navigation performance [19, 23, 38, 48]. Specifically, Saucier et al. [7] demonstrated that women outperformed men in the matrix navigation task when directions were phrased in egocentric and landmark-based terms. Note, however, that they used a 2D version of the task, for which sex differences are generally smaller [7, 12, 23].

Our results, however, show that the modulation of sex differences in navigation by perspective and strategy was not as straightforward as hypothesized.

Regarding perspective, both male and female participants needed more time to locate the target when directions were phrased in allocentric rather than egocentric terms. The egocentric perspective allowed for the fastest navigation, irrespective of sex and strategy. This is in contrast to findings by Saucier et al. [7], who found that men performed better with allocentric compared to egocentric directions. Note, however, that in their study, egocentric directions were confounding with a landmark-based strategy and allocentric directions with a Euclidian strategy. It is also possible that the favorable use of perspectives is shaped by education and experience. Previously, navigating via maps required the use of allocentric perspectives; nowadays, most car navigation system or online mapping services use the egocentrical perspective and Euclidian strategy to navigate; therefore, people are exposed to this type of instruction more frequently, whereas the allocentric instruction only constantly occurs in a specialized field such as the military [54–56].

However, sex differences are stronger for the allocentric compared to the egocentric perspective, i.e., the allocentric perspective impaired performance in women more than in men. This is particularly apparent when focusing on the Euclidian strategy. Thus, while navigation performance in men is not facilitated by using the allocentric perspective as suggested by the literature, it is certainly the case that men are less impaired by the allocentric perspective than women.

Regarding strategy, sex differences were stronger for the Euclidian than for the landmark-based strategy. However, a sex*strategy interaction was only observed for the allocentric perspective, but not for the egocentric perspective. For allocentric directions, women showed an advantage with a landmark-based strategy, while men showed an advantage with a Euclidian strategy as hypothesized. It is possible that for the egocentric perspective, a ceiling effect was achieved, such that strategy differences were not picked up by performance measures. Egocentrical instructions are easier to understand and learned earlier in life, making navigation universally easier compared to the specific training needed to master the allocentric perspective [54–59]. This is in line with the claim by Coluccia and Louse that sex differences tend to appear only when the task is difficult [24, 25].

Notably, these findings were mediated neither by confounding variables like ego-shooter experience and training effects nor by sex hormone levels. Thus, sex differences in virtual navigation performance cannot solely be explained by men’s higher experience with virtual worlds. The fact that the results were also irrespective of sex hormone levels suggests that these differences in performance and cognitive strategies are stable and develop either under the influence of organizational effects of sex hormones, due to socialization, or other factors that are irrespective of activational effects of sex hormones. It is however also possible that methodological factors, like power considerations or the non-invasive assessment of hormones from saliva, prevented us from detecting potential relations to sex hormone levels. Notably, we were able to detect menstrual cycle-dependent changes in perspective and strategy in a previous version of the task [40].

No sex differences were observed when identifying north. For this question, the allocentric perspective was beneficial compared to the egocentric perspective, irrespective of sex. The instructions for the allocentric perspective are phrased using the cardinal directions (“north,” “south,” “east,” “west”), giving participants obvious pointers along the way indicating in which direction they are currently moving, which makes the decision easier. With the egocentric instruction, participants only register the current cardinal direction once when they leave the starting block and have to remember in which direction they started. For reaction times, a significant advantage in identifying north was also observed with landmark-based instructions. This suggests that both men and women anchor cardinal directions at landmarks in the environment (e.g., the church is north), such that landmark information facilitates a sense of one’s position with respect to the cardinal directions in the environment. While these results were observed for both men and women, they also suggest that spatial orientation in a virtual environment is modulated interactively by perspective and strategy and that these two factors should not be confounded with each other.

In summary, this is the first study to adequately disentangle previously confounded roles of perspective and strategy for sex differences in the navigation task. The results are in line with previous findings but suggest that perspective and strategy modulate sex differences in navigation performance interactively rather than independently. Thus, the 3D navigation task created for this study is particularly valuable for research on different navigation strategies used by men and women.

## Footnotes

1 https://www.ncbi.nlm.nih.gov/pmc/articles/PMC5355435/ #fn01
